# Recombinant Enolase of *Trypanosoma cruzi* as a Novel Vaccine Candidate against Chagas Disease in a Mouse Model of Acute Infection

**DOI:** 10.1155/2018/8964085

**Published:** 2018-05-07

**Authors:** Minerva Arce-Fonseca, María Cristina González-Vázquez, Olivia Rodríguez-Morales, Verónica Graullera-Rivera, Alberto Aranda-Fraustro, Pedro A. Reyes, Alejandro Carabarin-Lima, José Luis Rosales-Encina

**Affiliations:** ^1^Departamento de Biología Molecular, Juan Badiano No. 1, Col. Sección XVI, Delegación Tlalpan, Instituto Nacional de Cardiología “Ignacio Chávez”, 14080 Mexico City, Mexico; ^2^Departamento de Biología Celular, Avenida Instituto Politecnico Nacional No. 2508, Col. San Pedro Zacatenco, Centro de Investigación y de Estudios Avanzados del Instituto Politecnico Nacional, 07360 Mexico City, Mexico; ^3^Departamento de Anatomía Patológica, Juan Badiano No. 1, Col. Sección XVI, Delegación Tlalpan, Instituto Nacional de Cardiología “Ignacio Chávez”, 14080 Mexico City, Mexico; ^4^Departamento de Infectómica y Patogénesis Molecular, Avenida Instituto Politecnico Nacional No. 2508, Col. San Pedro Zacatenco, Centro de Investigación y de Estudios Avanzados del Instituto Politecnico Nacional, 07360 Mexico City, Mexico

## Abstract

*Trypanosoma cruzi* is the protozoan parasite that causes Chagas disease, which is considered by the World Health Organization to be a neglected tropical disease. Two drugs exist for the treatment of Chagas disease, nifurtimox and benznidazole; they are only effective in the acute phase, and a vaccine is currently not available. In this study, we used the recombinant enolase from *T. cruzi* H8 strain (MHOM/MX/1992/H8 Yucatán) (rTcENO) and its encoding DNA (pBKTcENO) to immunize mice and evaluate their protective effects in an experimental murine model of acute phase infection. Our results showed that mice vaccinated with rTcENO or its encoding DNA were able to generate typical specific antibodies (IgG1, IgG2a, and IgG2b), suggesting that a mixed Th1/Th2 immune response was induced. The parasite burden in the blood was reduced to 69.8% and 71% in mice vaccinated with rTcENO and pBKTcENO, respectively. The group vaccinated with rTcENO achieved 75% survival, in contrast to the group vaccinated with pBKTcENO that showed no survival in comparison to the control groups. Moreover, rTcENO immunization elevated the production of IFN-*γ* and IL-2 after the parasite challenge, suggesting that the Th1-type immune response was polarized. These results indicated that rTcENO could be used as a vaccine against Chagas disease.

## 1. Introduction

The intracellular protozoan parasite *Trypanosoma cruzi* is the etiologic agent of Chagas disease, considered as a neglected tropical disease [1]. Currently, approximately 5.7 million people are infected worldwide, more than 70.2 million people are at risk of contracting the disease [2], and 50,000 patients die each year as a result of the disease [3].

Because of its natural life cycle, involving mitotic division in reduviid insects, which then transmit the infection by feeding on the blood of different vertebrates, *T. cruzi* is considered to be a severe health problem in rural areas of Mexico and Central and South America, where these insects are endemic. Chagas disease is also a health problem in nonendemic countries, such as the United States of America (USA), Canada, Australia, Japan, France, Spain, and Switzerland [4–8]. In Mexico, as well as in other endemic countries, cases are becoming more common in urban areas. This shift in the epidemiology of Chagas disease is connected to the migration of people infected with *T. cruzi*, blood transfusion, vertical transmission (mother to child), and organ transplantation [9].

Chagas disease has two different phases: acute and chronic. The acute phase is usually subclinical. Most acute cases are asymptomatic, last for 6–12 weeks, and typically occur in childhood [10]. Two major forms of the disease are observed during the chronic phase: indeterminate or latent and symptomatic. A high percentage of chagasic patients remain in the indeterminate phase for 10 or 30 years or even for life. These patients usually have no clinical or physical signs of disease but display positive serology. Approximately 30–40% of infected individuals develop clinical symptoms, involving severe cardiomyopathy or gastrointestinal pathology, several years after the infection.

Nifurtimox and benznidazole are currently the only licensed drugs with proven efficacy specifically against Chagas disease. Both drugs have significant activity during the acute phase of the disease, causing parasitological cure in up to 80% of patients who were treated early [11, 12]. In addition to chemotherapy, a vaccine may provide a suitable preventive measure to control the spread of the disease. Different parasite antigens have been tested for their effectiveness in controlling the infection. These immunizations, which were carried out at the level of DNA or recombinant proteins, showed different degrees of protection (e.g., reduced parasitemia and survival). In most immunization cases, a Th1-type immune response was observed. These genes and proteins were tested for vaccine trials in experimental models of infection, including cruzipain [13, 14], *trans*-sialidase [15, 16], amastigote surface protein-2 [17], LYT-1 [18], and paraflagellar rod protein [19] among others.

In recent years, several studies have shown that the “moonlighting” protein enolase is capable of generating a protective immune response against *Plasmodium falciparum* [20], *Candida albicans* [21], and *Ascaris suum* [22].

Enolase (2-phospho-d-glycerate hydrolase, EC 4.2.1.11) is a metalloenzyme that catalyzes the reversible dehydration of d-2-phosphoglycerate (PGA) to phosphoenolpyruvate (PEP) in both glycolysis and gluconeogenesis. Enolases, ranging from bacteria to higher vertebrates, show highly conserved amino acid sequences, particularly at the catalytic site. Consequently, enzymes from diverse species share similar kinetic properties. Enolase requires magnesium for both catalysis and dimer stabilization [23]. Furthermore, enolase that acts as a plasminogen receptor on the cell surface of certain pathogens [24, 25] has been implicated in nuclear functions, such as transcriptional regulation (as a repressor or activator) in protozoa [26–28], plants [29], and animal cells [30, 31]. Enolase is also involved in the stress response [32], vacuolar fusion processes [33], and alternative molecular chaperone functions [34, 35].

Previously, we cloned and sequenced the gene encoding enolase from *T. cruzi* and performed immunological in silico assays. Our data showed that the resulting sequence had several predicted peptides for B cells and cytotoxic T lymphocytes (CTL), which suggested that enolase could be a good immunogen [36].

In the present study, we immunized mice with the recombinant protein rTcENO or the recombinant pBKTcENO DNA plasmid. We then challenged the immunized mice with a lethal dose of *T. cruzi*. The mice immunized with rTcENO showed typical immunoglobulins for Th1/Th2 immune responses, a significant reduction in the level of parasite burden in the blood, and 75% survival rate in comparison to the control groups. Moreover, the detection of IFN-*γ*, TNF (alpha and beta), and IL-2, but not IL-4, showed a polarized Th1 immune response when mice were challenged with *T. cruzi*. Conversely, mice immunized with pBKTcENO did not survive after challenge with a high inoculum of *T. cruzi*, despite a significant reduction in the level of parasitemia. We found that immunization with rTcENO, but not with pBKTcENO, induced substantial protection in mice, indicating that rTcENO could be a good candidate to develop a vaccine against Chagas disease.

## 2. Materials and Methods

### 2.1. Immunization and *Trypanosoma cruzi* Challenge

All mice (female BALB/c mice 6–8 weeks old) were randomly assigned into control or vaccinated groups of eight mice each in two independent experiments. The mice were immunized by intraperitoneal (i.p.) injection with 10 *μ*g of the recombinant protein (rTcENO) emulsified in Freund's complete adjuvant (CFA) (Sigma) and boosted twice with 10 *μ*g of the rTcENO in Freund's incomplete adjuvant (IFA) every 2 weeks. The control, nonvaccinated animals were mock immunized with PBS/adjuvant in the same schedule as the immunized mice (they received one injection with CFA and two with IFA). This i.p. route has been used in the mouse model for immunization of vaccines based on recombinant proteins, epitopes, or microvesicles, demonstrating its protective efficacy [37–41].

For DNA-based immunizations, 100 *μ*g of recombinant plasmid (pBKTcENO) or vector DNA (pBK-CMV) (Stratagene) was dissolved in 50 *μ*L of sterile PBS, injected intramuscularly (i.m.) in the *tibialis anterioris* muscle and boosted twice every 2 weeks [42].

Both vaccinated (rTcENO or pBKTcENO) and control mice (PBS or pBK-CMV) had access to food and water ad libitum, and two weeks after the last immunization, they received an i.p. injection of 8 × 10^4^ bloodstream trypomastigotes of *T. cruzi*. The *T. cruzi* H8 strain (MHOM/MX/1992/H8 Yucatán (*T. cruzi*)) used in this work was a kind gift from Dr. Jorge E. Zavala Castro from the Centro de Investigaciones Regionales “Dr. Hideyo Noguchi”, Universidad Autónoma de Yucatán, Mérida, Yucatán, Mexico. Blood samples were collected from the tail vein to determine parasite burden every three days in the peripheral blood; in another similar experiment, survival rates were monitored daily. Mice were housed in a controlled environment and managed according to the National Institutes of Health Guide for Care and Use of Experimental Animals [43], with the approval of the CINVESTAV-IPN Animal Care and Use Committee.

### 2.2. rTcENO Polyclonal Antibodies

The recombinant protein rTcENO was obtained as previously described [36] and the purified rTcENO did not contain detectable levels of endotoxin contamination as measured by the E-Toxate assay (Sigma, St. Louis, MO, USA). Female BALB/c mice (6–8 weeks old) were immunized with10 *μ*g/mouse. Each animal received three i.p. doses of antigen every seven days; the first immunization dose was administered in complete Freund's adjuvant, and the following immunizations were administered in incomplete Freund's adjuvant. Before and at the end of the immunization scheme, animals were bled to collect serum.

### 2.3. Plasmid DNA Construction and Purification

The gene encoding TcENO (GenBank access number: KC862322) was obtained from the pRSETB-TcENO plasmid [36] as a 1.1 kb Kpn I/Hind III (New England Biolabs) fragment. This fragment was subcloned into the prokaryotic/eukaryotic expression vector pBK-CMV (InvitrogenTM by Life Technologies) to generate the pBKTcENO plasmid. The correct subcloning of the TcENO gene was confirmed by restriction enzyme analysis and sequencing.

Plasmid DNA was purified by anion-exchange chromatography using a Qiagen Plasmid Maxi Kit. DNA used for immunizations was sterilized by ethanol precipitation and resuspended in lipopolysaccharide-free PBS (Gibco).

Enolase was identified in *T. cruzi* total protein extracts or purified rTcENO by western blot.

Briefly, *T. cruzi* epimastigotes were harvested from cultures and resuspended in lysis buffer (10 mM Tris-HCl, pH 7.5; 5 mM EDTA; 1% Nonidet P-40; 1 mM phenyl-methanesulfonyl fluoride; 10 mg/mL aprotinin; 50 U/L trasylol; and 10 mg/mL leupeptin) by repeated freezing and thawing cycles. Lysates were cleared by centrifugation (30 min, 4°C at 14,000 ×g), and the supernatants were collected and resolved by 12% sodium dodecyl-sulfate polyacrylamide gel electrophoresis (SDS-PAGE, 10 *μ*g and 20 *μ*g per lane) or purified rTcENO (10 *μ*g per lane). Proteins were electrotransferred onto nitrocellulose membranes at 70 V for 1 h. The membranes were blocked with 5% (*w*/*v*) skim milk in phosphate-buffered saline (PBS, pH 7.4) for 1 h at 37°C, washed three times with PBS containing 0.05% Tween 20 (PBS-T), 10 min/per time, and then incubated overnight at 4°C with anti-rTcENO polyclonal antibodies (1 : 500 dilution in 2% skim milk-PBS) or anti-pBKTcENO polyclonal antibodies (1 : 500 dilution in 2% skim milk-PBS). The negative control consisted of a pool of serum from different healthy mice diluted 1 : 500 in PBS with 2% nonfat milk. After washing, the membranes were incubated with secondary antibody conjugated with alkaline phosphatase (Zymed Lab) for 1 h at 37 ° C at 1 : 5000 dilution in 2% skim milk-PBS. The blots were visualized with nitro-blue tetrazolium chloride (NBT) and 5-bromo-4-chloro-3′-indolyphosphate p-toluidine salt (BCIP) (Sigma).

### 2.4. Indirect Immunofluorescence Assays

*T. cruzi* epimastigotes were washed three times in PBS supplemented with 0.1% glucose (PBSG), pH 7.4, and fixed in 2% paraformaldehyde in PBS (*v*/*v*) for 2 h at 4°C. After that, 1 × 10^6^ parasites/mL were placed on glass slides for 45 min at 37°C. These preparations were divided into two sets; one remained nonpermeabilized, and the other was permeabilized with 0.2% Triton X-100 in PBS. Both preparations were incubated with antibodies against either TcENO or *T. cruzi* total protein extract from mice serum (diluted 1 : 100 in PBS-5% BSA) for 1 h at room temperature using an FITC-labelled IgG (Pierce) as a secondary antibody diluted 1 : 3000 in PBS. After 1 h at room temperature, we rinsed the slides in PBS, stained the nuclear and kinetoplast DNA with DAPI, and mounted them with Vecta-Shield medium (Vector Laboratories). The results were observed with a Carl Zeiss LSM 700 confocal microscope.

### 2.5. Immunoglobulin Determination

Total IgG immunoglobulin and isotypes IgG1, IgG2a, IgG2b, IgG3, IgA, and IgM were evaluated by the ELISA method according to the manufacturer's instructions (Zymed Labs). Briefly, plates were coated with rTcENO (2 *μ*g/mL) in carbonate buffer (pH 9.6) and incubated overnight at 4°C. Plates were washed with PBS-0.1% Tween (PBST), incubated for 2 h at 37°C with blocking solution (PBS containing 5% skim milk), washed with PBST and PBS, and then incubated with 50 *μ*L of either mouse anti-rTcENO or anti-pBKTcENO (1 : 500 dilutions). As a negative control, a pool of preimmune sera was used in all experiments. After washing, the peroxidase-labeled rabbit anti-mouse IgG antibodies were added at 1 : 1000 dilution in PBST and incubated for 1 h at room temperature. Plates were incubated with 100 *μ*L of ABTS substrate (2,2,-azino-bis[3-ethylbenzthiazoline]-6-sulphonic acid) (Zymed Labs) for 10 min and read at 405 nm in an ELISA microplate reader (MultisKan MS).

### 2.6. Cytokine Determination by Flow Cytometry

The levels of serum Th1- (INF-*γ*, IL-2, and TNF) and Th2- (IL-4) type cytokines were analyzed in duplicate using a fluorescent bead immunoassay for quantitative detection by flow cytometry (Mouse Th1/Th2 Cytokine Kit, BD™ Cytometric Bead Array (CBA); BD Biosciences) according to the manufacturer's instructions. Briefly, serum samples (50 *μ*L) and phycoerythrin- (PE-) conjugated antibodies were incubated with capture bead reagent for 2 h in the dark at room temperature. Unbound antibodies were washed (1.0 mL wash buffer) and resuspended in 300 mL of PBS. Samples were analyzed in a FACScalibur Flow Cytometer (BD Biosciences), and fluorescence intensity was calculated using FCAP Array v3 Software (BD Biosciences). All four cytokines exhibited single well-separated peaks. Four individual cytokine standard curves (range 20–5000 pg/mL) were run in each assay. Cytokine concentrations were determined by reference to standard curves and expressed in pg/mL.

### 2.7. Histology

Heart and skeletal muscle samples were aseptically isolated, rinsed with sterile PBS, and fixed in 4% paraformaldehyde in PBS (pH 7.4) for 24 h. Fixed samples were embedded in paraffin, sectioned (5 *μ*m), stained with hematoxylin and eosin or Masson trichrome, and examined by light microscopy (Nikon Eclipse E600). To examine inflammatory infiltrate/myocarditis or myositis and amastigote nets, each tissue section was analyzed for >10 microscopic fields (40x magnifications) by two investigators who were blinded to identity of the experimental groups. Myocarditis or myositis (presence of inflammatory cells) from H&E-stained sections was scored as 0 (absent, without foci of inflammation), 1 (1 or less foci of inflammatory cells/field), 2 (moderate, >2 foci/field), 3 (generalized coalescing of foci of inflammation or disseminated inflammation with minimal cell necrosis and retention of tissue integrity), and 4 (diffused inflammation with severe tissue necrosis, interstitial edema, and loss of integrity).

### 2.8. Statistical Analysis

The results were expressed as the means ± SD. Statistical analysis was performed using one-way ANOVA followed by Tukey's test. The survival time was calculated by the Kaplan-Meier method. Data sets that were found not to be normally distributed were analyzed with the Kruskal-Wallis test followed by the Mann–Whitney test to assess the differences between pairwise comparisons. Differences were considered to be statistically significant when the *p* value was <0.01 or <0.05.

## 3. Results

### 3.1. Polyclonal Antibodies Anti-TcENO Are Specific

**Figure 1:**
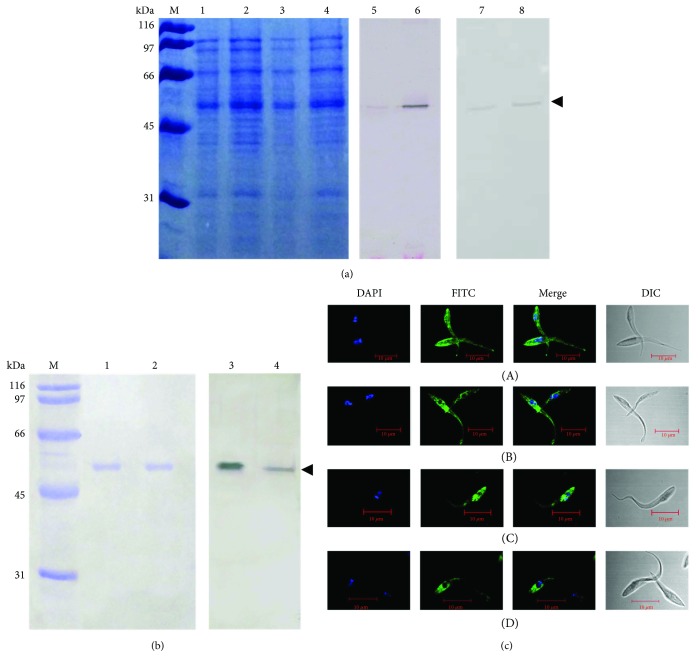
*T. cruzi* enolase immunodetection. (a) Total extract of *T. cruzi* soluble proteins and its respective replica for western blotting. Lane M: molecular weight marker; lanes 1 and 3: total soluble proteins (10 *μ*g); lanes 2 and 4: total soluble proteins (20 *μ*g); lanes 5 and 6: western blot of immobilized proteins with polyclonal antibodies anti-rTcENO; lanes 7 and 8: western blot of immobilized proteins with polyclonal antibodies anti-pBKTcENO. (b) Purified rTcENO and its respective replica for western blotting. Lane M: molecular weight marker; lanes 1 and 2: rTcENO (10 *μ*g); lane 3: western blot with pool of polyclonal antibodies anti-rTcENO; lane 4: western blot with pool of polyclonal antibodies anti-pBKTcENO. The arrowhead indicates the signal for a band of approximately 46 kDa (the TcENO estimated weight). (c) Indirect immunofluorescence assay. The secondary antibody that recognized the anti-rTcEno was FITC labeled (green fluorescence). Nuclear and kinetoplast DNA were stained with DAPI and shown by blue fluorescence. Each image is a representative of at least two independent experiments and captured by confocal microscope. (A) Nonpermeabilized parasite with anti-*T. cruzi* polyclonal antibodies; (B) nonpermeabilized parasite with anti-rTcENO polyclonal antibodies; (C) permeabilized parasite with anti-*T. cruzi* polyclonal antibodies; (D) permeabilized parasite with anti-rTcENO polyclonal antibodies.

In our previous work, we cloned the enolase gene sequence into the pRSETB vector and purified the recombinant rTcENO protein [36]. In this report, we immunized mice with the recombinant rTcENO protein to produce polyclonal antibodies. The endotoxin level of rTcENO used for immunization was found to be <1 EU/mL. The serum of these immunized mice was used in western blot assays as a first antibody against *T. cruzi* total protein extract, resulting in a specific band of approximately 46 kDa. This band was also detected when anti-pBKTcENO antibodies were used (Figure 1(a)). To confirm the specificity of the antibodies generated by immunization with rTcENO and pBKTcENO, the antibodies produced were used in western blot assays against purified rTcENO. Both antibodies recognized the recombinant protein, indicating that the antibodies generated were specific (Figure 1(b)).

The antibodies against rTcENO were also used in immunofluorescence assays against permeabilized and nonpermeabilized *T. cruzi* cells. In permeabilized parasites, TcENO labeling was found in the cytoplasm, and no reaction was detected in structures such as the flagellum, whereas in nonpermeabilized parasites, labeling was found in the membrane. This localization might facilitate its recognition by the host's immune system, suggesting that TcENO could have strong antigenic properties (Figure 1(c)).

### 3.2. Humoral Response in Mice Immunized with rTcENO or pBKTcENO

**Figure 2:**
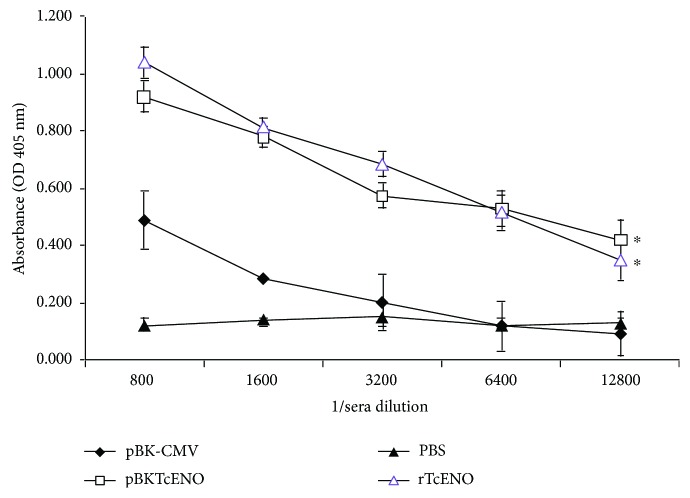
Antibody titers in immunized mice. ELISA was performed seven days after the last immunization to evaluate the serum levels (absorbance in optical density at 405 nm) of rTcENO-specific antibodies at different dilutions. The values represent the average of triplicate assays ± S.D. A significant difference was detected by comparing rTcENO or pBKTcENO versus PBS or pBK-CMV (^∗^*p* < 0.01).

**Figure 3:**
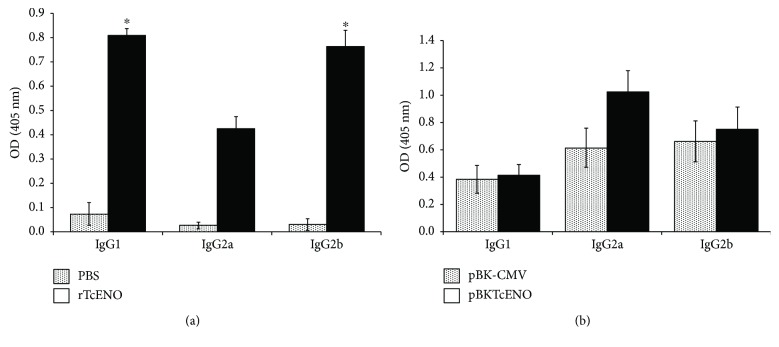
Antibody isotypes in immunized mice. Seven days after the last immunization with rTcENO (a) or pBKTcENO (b), generated antibody isotypes were evaluated by ELISA. The plotted data show optical density (OD) values for eight mice per group representing the mean ± SD of at least three independent experiments. A significant difference was detected in immunized mice with rTcENO by comparing IgG1 and IgG2b versus IgG2a (^∗^*p* < 0.01).

To characterize the immune response induced by *T. cruzi* enolase, we immunized BALB/c mice intraperitoneally with rTcENO or intramuscularly with pBKTcENO as described in Materials and Methods. The immunizations with either rTcENO or pBKTcENO induced a significant production of IgGs against the rTcENO antigen (Figure 2) seven days after the last immunization. Moreover, antigen-specific isotypes of immunoglobulins in the sera of immunized animals with rTcENO revealed high levels of IgG1>IgG2b>IgG2a (Figure 3(a)), indicating that this antigen induced a mixed Th1-/Th2-like immune response. In contrast, the mice immunized with pBKTcENO showed IgG2a>IgG2b>IgG1 with an IgG2b/IgG1 ratio > 1, suggesting that a predominantly Th1-like immune response was induced (Figure 3(b)). As expected, the mice immunized with rTcENO or pBKTcENO exhibited high titers of antibodies compared to the control groups inoculated with PBS or pBK-CMV (*p* < 0.01). In contrast, the isotyping mice immunized with pBKTcENO did not show any significant difference from those in the pBK-CMV group.

### 3.3. rTcENO or pBKTcENO Immunizations Reduce the Parasitemia, but Only Mice Immunized with rTcENO Survive after Challenge with *T. cruzi*

**Figure 4:**
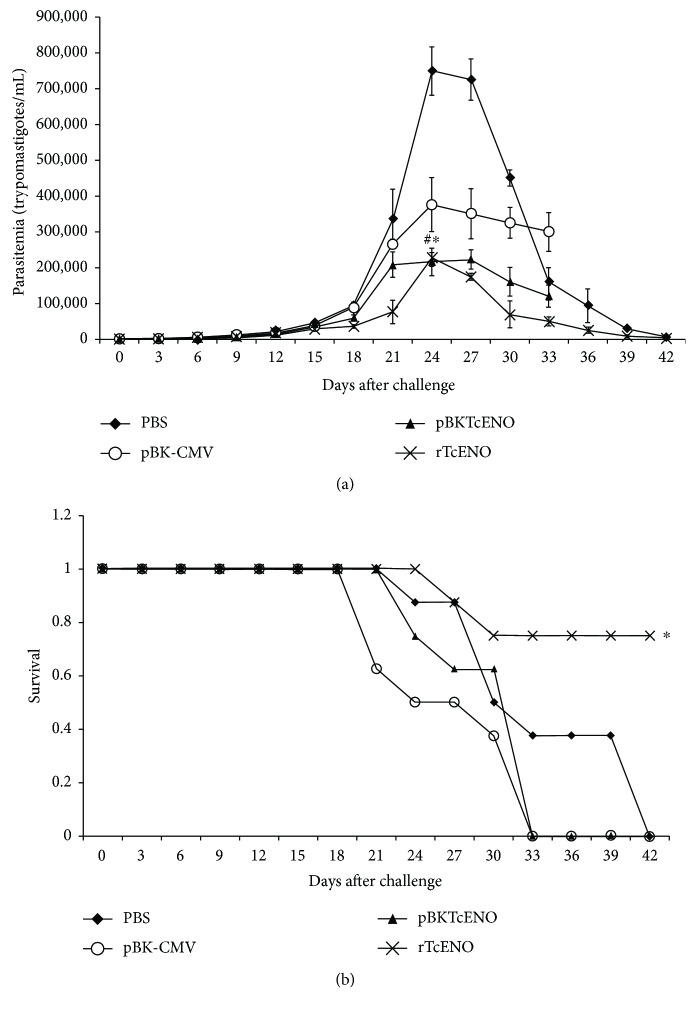
Evaluation of protection generated by vaccination with rTcENO or pBKTcENO. (a) Parasitemia in immunized mice after challenge with *T. cruzi*. BALB/c mice were immunized as described in Materials and Methods. Values plotted show the mean ± standard deviation of eight mice per group and are representative of two independent experiments. At the peak of infection, parasitemia levels were compared using one-way analysis of variance and post hoc Tukey's tests. A significant difference was detected by comparing rTcENO versus PBS (^∗^*p* < 0.01), pBKTcENO versus PBS (^#^*p* < 0.01) and pBKTcENO versus pBK-CMV (^∗^*p* < 0.01). (b) Survival of immunized and infected mice. Values plotted show the mean ± SD of eight mice per group and are representative of two independent experiments with similar results. A statistically significant difference (^∗^*p* < 0.01) between rTcENO versus PBS is indicated.

To determine if immunizations with either rTcENO or pBKTcENO reduce parasitic burden in the blood and confer protection to experimentally infected mice with *T. cruzi*, we recorded parasitemia profiles and mortality rates. The parasitemia profile showed a 69.8% decrease when the mice were immunized with rTcENO compared to the control group (PBS) (*p* < 0.01) (Figure 4(a)). The pBKTcENO plasmid also reduced the parasite load in the mice at 70% and 42% during the parasitemia peak (day 24 after challenge) compared to the PBS and pBK-CMV controls, respectively (*p* < 0.01).

The mice immunized with rTcENO showed 75% survival rate compared to the control group (PBS) (*p* < 0.01) at the end of the experiment. Nevertheless, all mice immunized with pBKTcENO eventually died, not exceeding day 33 postinfection (Figure 4(b)).

### 3.4. Th1-Type Immune Response Is Polarized in rTcENO-Immunized Mice after Parasite Challenge

**Figure 5:**
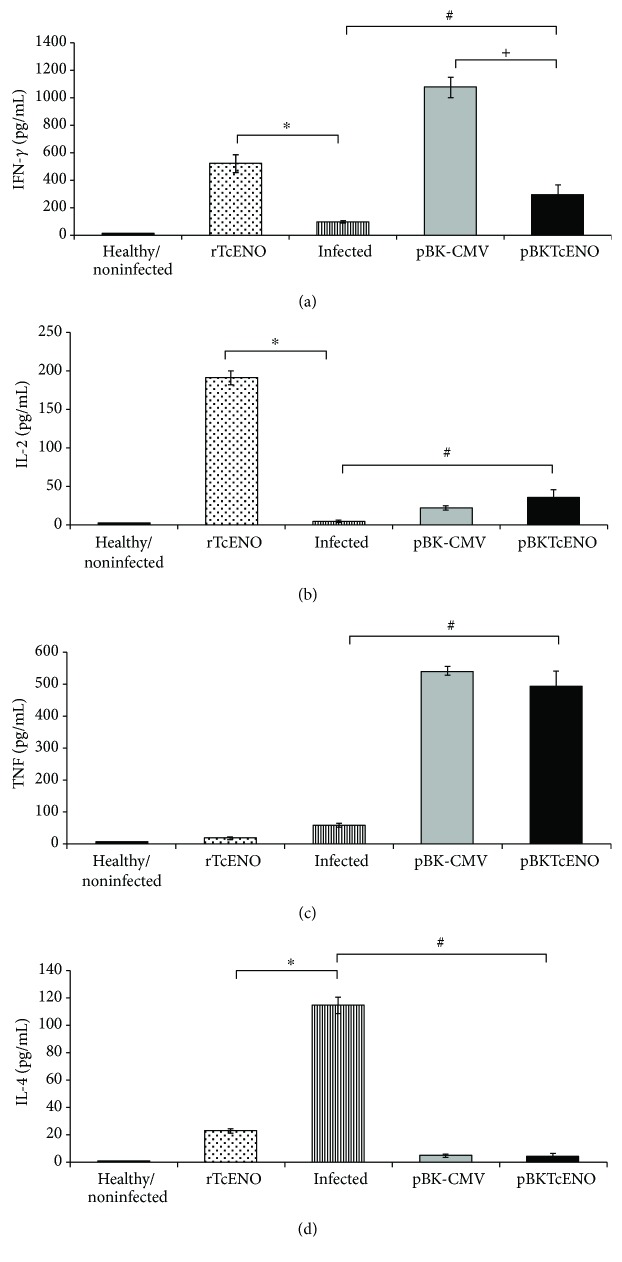
Serum cytokine levels in immunized and nonimmunized mice after challenge. Serum levels of (a) IFN-*γ*, (b) IL-2, (c) TNF, and (d) IL-4 were determined by flow cytometry. The concentration of a particular cytokine was established by comparing the obtained data with a standard curve for each experiment. The data are represented as the mean ± SD. All measurements were performed in duplicate, with serum from eight mice per group. Sera from healthy mice (noninfected and nonimmunized) were used as controls. Significant differences were detected as follows: rTcENO versus infected (^∗^*p* < 0.01), pBKTcENO versus infected (^#^*p* < 0.01), and pBKTcENO versus pBK-CMV (^+^*p* < 0.01).

To determine the type of immunological response induced after parasite challenge, the mice were bled after the peak of parasitemia (day 30 after challenge), and serum IFN-*γ*, IL-2, TNF, and IL-4 cytokines were determined (Figure 5). The mice vaccinated with rTcENO showed increased levels of the Th1-related cytokines IFN-*γ* and IL-2 but low levels of TNF compared with the nonvaccinated control group (*p* < 0.01). In contrast, the mice vaccinated with pBKTcENO showed significantly higher levels of TNF and IFN-*γ* and lower levels of IL-2, but these latter levels were significantly higher than those found in nonvaccinated mice. The levels of TNF in the control mice vaccinated with the plasmid pBK-CMV were higher than those in the mice vaccinated with pBKTcENO (*p* < 0.01).

### 3.5. Immunization with rTcENO Confers Protection to the Heart and Skeletal Muscle

**Figure 6:**
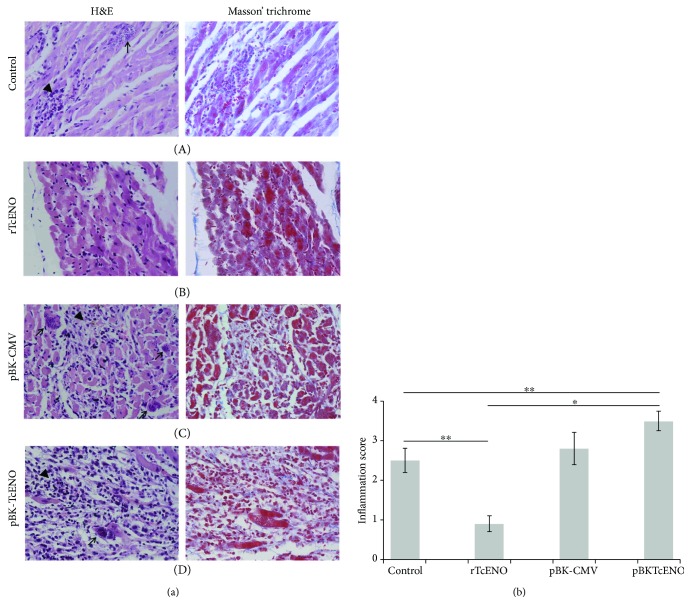
Vaccination effects on the cardiac muscle sections. (a) Tissue sections were stained with hematoxylin and eosin or Masson's trichrome and visualized by light microscopy (original magnification: 40x). Representative micrographs of the heart tissue from control-infected mice and mock-immunized with PBS (A), rTcENO (B), pBK-CMV empty plasmid vector (C), and pBK-TcENO (D) are shown. Black arrows show amastigote nests. Black arrowheads show inflammatory infiltrates. (b) Inflammatory lesion (lymphocyte infiltrates) scores. The inflammatory score was derived from two different experiments as described in Materials and Methods. The data are expressed as mean ± SD, and significance is presented as ^∗^*p* < 0.01 and ^∗∗^*p* < 0.05 (^∗∗^control group nonimmunized and infected versus vaccinated and infected groups, rTcENO and pBK-TcENO; ^∗^rTcENO versus pBK-TcENO).

Histological analysis of the heart of nonvaccinated mice and that of mice immunized with pBK-CMV revealed histopathological features compatible with acute chagasic myocarditis. We observed numerous nests of *T. cruzi* amastigotes accompanied by inflammatory infiltrates and few affected myocardial fibers that showed regeneration. In contrast, histological analysis of the heart of mice immunized with rTcENO revealed diffuse myocarditis with a mild mononuclear inflammatory infiltrate composed mainly of lymphocytes and a few plasma cells, while amastigote nests were absent. In mice immunized with pBKTcENO, severe myocarditis, with the presence of amastigote nest associated with necrosis of the myocardial fibers, and intense inflammatory infiltrates, composed of lymphocytes, plasma cells, and a few polymorphonuclear leukocytes, were observed (Figure 6).

**Figure 7:**
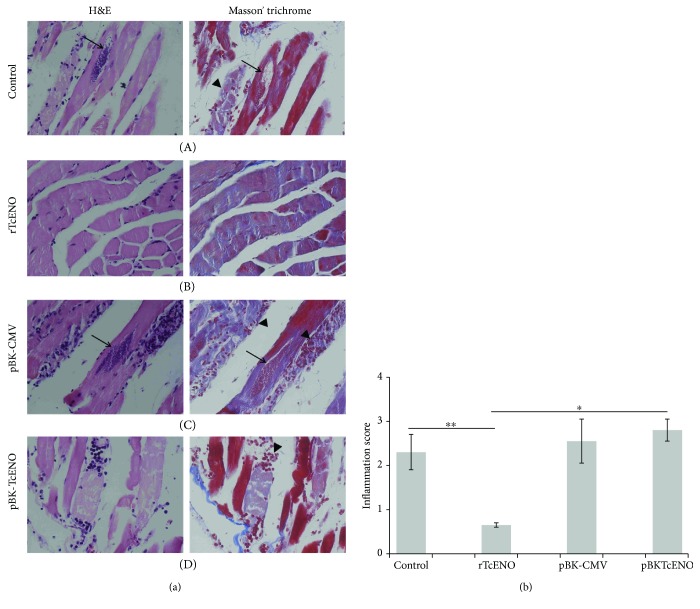
Vaccination effects on the skeletal muscle sections. (a) Tissue sections were stained with hematoxylin and eosin or Masson's trichrome and visualized by light microscopy (original magnification: 40x). Representative micrographs of skeletal tissue from control-infected mice and mock-immunized with PBS (A), rTcENO (B), pBK-CMV empty plasmid vector (C), and pBK-TcENO (D) are shown. Black arrows show amastigote nests. Black arrowheads show inflammatory infiltrates. (b) Inflammatory lesion (lymphocyte infiltrates) scores. The inflammatory score was derived from two different experiments as described in Materials and Methods. The data are expressed as mean ± SD, and significance is presented as ^∗^*p* < 0.01 and ^∗∗^*p* < 0.05 (^∗∗^control group nonimmunized and infected versus vaccinated and infected groups, rTcENO and pBK-TcENO; ^∗^rTcENO versus pBK-TcENO).

Skeletal muscle samples of the control mice exhibited disorganization of tissue architecture, necrosis, and large nests of *T. cruzi* amastigotes, while in the vaccinated mice, the skeletal muscle showed inflammatory infiltrates that consisted mainly of mononuclear cells. We did not detect any amastigote nests in the mice vaccinated with rTcENO (Figure 7).

## 4. Discussion

Chagas disease is a neglected tropical disease that affects the poorest population in endemic areas. Recently, several forms of transmission have reemerged and affected the population in nonendemic countries [9, 44]. Specific treatment of this disease consists of two drugs, nifurtimox and benznidazole. The efficacy of both drugs in the acute phase has been shown; however, their use in the chronic phase is currently the subject of discussion. Unfortunately, a vaccine for Chagas disease is not available to date [11, 45].

DNA vaccination has been shown to generate both humoral and cell-mediated immune responses [46] and has been shown to be an effective means of generating protective responses against *T. cruzi* infection in murine models [15, 42, 47]. Moreover, immunization with recombinant proteins has also generated promising results [16, 19, 48, 49]. Several membrane proteins have been proposed as good options to develop vaccines against different diseases. Among these membrane proteins, enolase has been demonstrated to be immunogenic [20–22] suggesting that a vaccine with this protein is possible. In our previous work, we evaluated the immunogenic characteristics of *T. cruzi* enolase using in silico assays. Our study showed that the recombinant protein was recognized by serum from both mice and humans infected with *T. cruzi* [36]. To support these data, in this study, we used rTcEno polyclonal antibodies against total protein extracts of *T. cruzi* in western blot and immunofluorescence assays to determine the specificity of these antibodies. These results demonstrated that enolase was located in both the cytoplasm and the cell membrane, in agreement with studies carried out in parasites such as *Echinococcus granulosus*, *Trichomonas vaginalis*, and *Plasmodium falciparum* [20, 50, 51].

Antibodies against both rTcENO and pBKTcENO were bound to intact epimastigotes, indicating that enolase protein was antigenic and exposed on the parasite surface.

Nevertheless, the exact mechanism by which enolase is secreted and translocated to the parasite surface is still unknown. However, it is known that the amino acid sequence lacks a conventional N-terminal signal sequence. It has been suggested that enolases could be secreted in exosomes and other vesicles [52] and that secreted enolases could reassociate with the cell membrane [24].

Enolase has been analyzed as a vaccine in several parasites and bacteria, demonstrating that it may be an important immunogenic protein and a protective antigen [20–22, 53–55]. In this study, analysis of the humoral immune response showed that the generated antibodies in the mice vaccinated with rTcENO were a mixture of Th1- and Th2-type immune responses (IgG1>IgG2b>IgG2a). Moreover, the mice vaccinated with pBKTcENO showed an increase in IgG2a immune response and the rate of IgG2b/IgG1 being >1, which suggested the dominance of Th1-type immune response. Previous studies showed that Th1-type immune response was required for clearing the parasite from infected mice [56, 57].

We found that the mice immunized with rTcENO were capable of significantly inducing IL-2 and IFN-*γ* in comparison to the control group, showing that in the immune response after challenge, there was a polarization towards Th1-type immune response. In the mice vaccinated with pBKTcENO, there was induction of IFN-*γ* and TNF but not IL-2. IFN-*γ* was required to activate macrophages and indirectly constitutes an important source of protective proinflammatory cytokines, which could effectively kill intracellular parasites such as *T. cruzi* by nitric oxide- (NO-) dependent mechanisms [58]. In this experimental group, discrete levels of IFN-*γ* were detected, and animals showed reduction in parasitemia profile. However, no mice survived at day 33 postchallenge. Interestingly, the pBK-CMV-immunized animals were able to induce high levels of IFN-*γ* and decrease parasitemia significantly compared to the PBS control group. Despite its low parasitemia, this group also showed reduced survival. These results might be due to the induced response by immunostimulatory sequences in the plasmid that trigger innate immunity in the host [59] but could not confer a specific response that favors the survival of the animals against parasitic challenge.

Therefore, other factors that did not prevent mortality might be involved in the reduction of parasitemia. One of these factors could be the IL-10 cytokine because it acted as an immunoregulatory cytokine in the Th1-type response [60]. In turn, IL-10 would prevent the collateral damage generated by a strong immune response against the parasite and suppress the development of inflammatory cell infiltrates that otherwise would be exacerbated. A previous study proposed that an exacerbated response to infections could result in harmful injuries [61].

To determine whether vaccination conferred protection in the cardiac and skeletal muscle, histological sections were prepared and stained. The results showed that animals immunized with rTcENO were able to prevent the establishment of parasites in the heart tissue, as shown by the absence of amastigote nests and low amount of inflammatory infiltrates in the heart sections (Figures 6 and 7). Furthermore, the low amount of inflammatory cells indicated that the immune response was adequate to eliminate the parasites.

Although the pBKTcENO vaccine reduced the parasitemia in the immunized/infected mice, these animals showed exacerbated damage in the heart and skeletal tissues (Figures 5 and 6), with many amastigote nests and inflammatory cells. The tissue injured by *T. cruzi* typically shows mononuclear cells and progression from multifocal to diffuse lesions. Activated neutrophils and eosinophils are efficient cells to destroy *T. cruzi*, and indirectly their activity causes severe damage to other host cells. Although neutrophils and eosinophils are not as abundant as mononuclear cells, their presence correlates with the severity of lesions observed in the cardiac tissue [62, 63].

Several researchers have demonstrated that DNA immunization is effective in protecting animals against *T. cruzi*, particularly with *TSA-1* [64], *Tc2* [65], and *SSP4* [66] genes, where immunizations generated high levels of cytokines which modulate the Th1-type immune response such as IFN-*γ*, and in the case of *SSP4*, the immunization also favored the increase of IL-10.

The exact mechanism by which pBKTcENO-immunized/infected or pBK-CMV-mock-immunized/infected mice died after challenge with *T. cruzi* even after producing antibodies related to Th1-type immune response is not known. It is possible that these antibodies were not opsonic, failing to induce *T. cruzi* killing by mouse phagocytes, as it was reported by Esgleas et al. [67], who found that the immunization with SsEno of *Streptococcus suis* failed to protect mice after experimental infection with bacteria. Another possible explanation is that the immune response was exacerbated enough in the cardiac tissue to induce death of the immunized animals. In this case, IL-10 is an important pleiotropic immunoregulatory cytokine that can participate during the Th1 immune response [60] to prevent collateral damage generated by a strong immune response against the parasites and suppress the development of inflammatory cell infiltrates that otherwise would be exacerbated. However, IL-10 can suppress immune responses [68] thus favoring the observed increased levels of parasites after acute infection. Therefore, we will conduct experiments using anti-IL-10 mAbs or IL-10 KO mice to determine the role of IL-10 in the protection against death of vaccinated mice with pBKTcENO.

In summary, we have shown that the immunization with rTcENO effectively controlled acute *T. cruzi* infection in a murine model by reducing parasite burden in the blood, preventing the establishment of inflammatory infiltrates in the heart and skeletal muscle and increasing the survival of immunized mice. Our data support further studies to improve the efficacy of rTcENO and examine its potential as a good candidate for the development of a vaccine against *T. cruzi*.

## Data Availability

The data used to support the findings of this study are available from the corresponding author upon request.
